# Using Complexity-Identical Human- and Machine-Directed Utterances to Investigate Addressee Detection for Spoken Dialogue Systems

**DOI:** 10.3390/s20092740

**Published:** 2020-05-11

**Authors:** Oleg Akhtiamov, Ingo Siegert, Alexey Karpov, Wolfgang Minker

**Affiliations:** 1 Institute of Communications Engineering, Ulm University, Albert-Einstein-Allee 43, 89081 Ulm, Germany; wolfgang.minker@uni-ulm.de; 2 ITMO University, Kronverksky Ave. 49, 197101 St. Petersburg, Russia; 3 Institute for Information Technology and Communications, Otto-von-Guericke-University, Universitaetsplatz 2, 39016 Magdeburg, Germany; 4 St. Petersburg Institute for Informatics and Automation of the Russian Academy of Sciences, 14th Line 39, 199178 St. Petersburg, Russia; karpov_a@mail.ru

**Keywords:** addressee detection, human-computer interaction, computational paralinguistics, speaking style, data augmentation, mixup, speech classification

## Abstract

Human-machine addressee detection (H-M AD) is a modern paralinguistics and dialogue challenge that arises in multiparty conversations between several people and a spoken dialogue system (SDS) since the users may also talk to each other and even to themselves while interacting with the system. The SDS is supposed to determine whether it is being addressed or not. All existing studies on acoustic H-M AD were conducted on corpora designed in such a way that a human addressee and a machine played different dialogue roles. This peculiarity influences speakers’ behaviour and increases vocal differences between human- and machine-directed utterances. In the present study, we consider the Restaurant Booking Corpus (RBC) that consists of complexity-identical human- and machine-directed phone calls and allows us to eliminate most of the factors influencing speakers’ behaviour implicitly. The only remaining factor is the speakers’ explicit awareness of their interlocutor (technical system or human being). Although complexity-identical H-M AD is essentially more challenging than the classical one, we managed to achieve significant improvements using data augmentation (unweighted average recall (UAR) = 0.628) over native listeners (UAR = 0.596) and a baseline classifier presented by the RBC developers (UAR = 0.539).

## 1. Introduction

Spoken dialogue systems (SDSs) appeared a couple of decades ago and have already become part of our everyday life. Speech is the most natural way of communication between people, and therefore they usually prefer speech-based user interfaces over textual and graphical input alone when it comes to natural interaction with technical systems [1]. Speech input alone is particularly convenient for specifying abstract actions or defining multiple entities [2]. Sometimes a combination with additional modalities, usually touch or gesture, is preferred to resolve deictic references or spatial information [3,4]. In recent years, considerable progress has been made towards adaptive SDSs [5] and understanding multiparty conversations [6,7,8]. Virtual assistants, e.g., Siri, Cortana, Alexa, and Alisa, are typical examples of modern SDSs. Such systems face the problem of human-machine addressee detection (H-M AD) that arises in multiparty spoken conversations between several people and an SDS since the users may also talk to each other and even to themselves while interacting with the system [9,10]. The SDS is supposed to adapt to this mixed interaction, i.e., to determine whether the system is being addressed or not. The currently preferred approach to AD is confined to the detection if the user has pronounced a wake-word. Unfortunately, this method is too unnatural and error-prone to be used for realistic spoken interaction and often leads to some misunderstanding between SDSs and users. It is just annoying if the SDS is not activated when the wake-word has been said and the user has to utter the wake-word repeatedly. Sometimes, even worse, the system is activated due to a misunderstood phrase or due to the usage of the wake-word in a different context without any intention to interact with the SDS [11,12,13].

In the present study, we tackle H-M AD as a computational paralinguistics challenge and focus specifically on vocal accommodation. Such an approach to H-M AD does not require any visual, lexical, or language-specific information and therefore appears to be the most flexible solution that may theoretically be applied to various dialogue domains and languages [14]. The approach is based on the observation that people accommodate their manner of speech, making it more rhythmical, louder, and generally easier to understand, as soon as they start talking to an SDS, since they do not perceive the system as an adequate conversational partner [15]. Future users are unlikely to accommodate their manner of speech to SDSs to such a high degree as they do it presently, since modern systems are becoming more adaptive and human-like, especially in their ability to understand. Therefore, the prosody and complexity of a conversation depend on the communicational abilities of the user’s conversational partner. In this connection, we introduce the term “complexity-identical”. This term denotes conversations between a human and another human (H-H) and conversations between a human and a the technical device (H-M) with the same complexity in the sense of grammatical phrases. In such conversations, the user’s human-directed and machine-directed utterances are thus likely to be pronounced similarly.

AD problems and paralinguistic problems in general, are known to be highly language-, corpus-, and even speaker-dependent [16,17,18]. Nevertheless, existing studies on acoustic H-M AD use self-recorded data sets as there is no benchmark data set available [9,14,19]. Among the prominent INTERSPEECH challenges of recent years, there was just a single addressee sub-challenge in 2017 aimed at distinguishing adult-directed and child-directed utterances both produced by adults [20].

The authors of [21,22] trained their deep neural networks (DNNs) for acoustic H-M AD on huge corpora containing several hundred hours of speech data. However, we do not have that much data at our disposal. In this light, the present study has the following contributions. We investigate the dependence of acoustic models on various German H-M AD corpora, conducting a series of cross-corpus experiments. Applying a novel mixup-based algorithm, we augment the Restaurant Booking Corpus (RBC) of complexity-identical human- and machine-directed utterances [23] with other two corpora developed for classical H-M AD scenarios [9,24]. Using the obtained data mixture, we train an ensemble of classifiers combining advantages of both classical models and DNNs. The mixup technique has already been studied on image classification [25], speech recognition [26], and acoustic H-M AD [27]. As a result, we obtain the first competitive RBC baseline using cross-corpus data augmentation that significantly outperforms native listeners and an existing baseline classifier presented in [23] on the RBC classification problem.

We dedicated our previous study [27] to multitask learning and general mixup capabilities towards acoustic H-M AD, while the present research considers a similar but more challenging problem under some constraints that allow us to exclude collateral factors potentially influencing users’ acoustic behaviour. Compared to [27], the key novelty of the present paper is the comparison between the new complexity-identical RBC experimental setup and the classical experimental setup used for modelling H-M AD problems. RBC is introduced in the present paper and was not used in [27]. Another improvement is the ensemble of classifiers, while in [27] they were employed individually. Finally, the main focus of the present paper is to improve the classification performance on the most challenging corpus (RBC), while the paper [27] was focused on the multitask training of individual classifiers learning several corpora at once and on improving their multitask performance.

The paper is organised as follows: in Section 2, we analyse both classical and complexity-identical setups for modelling the H-M AD problem and briefly describe several existing studies using the classical H-M AD setup; in Section 3, we introduce speech data augmentation and classification methods; in Section 4, we describe three corpora, on which we conduct a series of experiments and report their results in Section 5. Finally, we make concluding remarks in Section 6.

## 2. Related Work

Most existing studies on acoustic H-M AD [9,14,19,28] are focused on acoustic feature design rather than on model design. Meanwhile, any additional data preprocessing results in information losses, and therefore data-driven and particularly end-to-end models based on DNNs dealing with low-level features can improve acoustic H-M AD. Solving the classical problem of acoustic H-M AD, the authors of [21] designed a huge DNN comprising multiple layers of Long Short-Term Memory (LSTM) cells to detect device-directed utterances. The best performing network configuration had around 16 million parameters and was trained on 280 h (238k device-directed and 162k non device-directed utterances) of real recordings of natural human interactions with voice-controlled far-field devices. More details on the used data set are not given. The network reached an equal error rate (EER) of 10.9%. In their next study [29], the researchers extended this model to have a separate output branch for each dialogue type resulting in a relative 12.5% of EER reduction. However, neither this network nor that one proposed in [22] were end-to-end since they both received log filter-bank features as the input for H-M AD. In addition to handcrafted features, we want our model to learn its own feature representations directly from raw waveforms. We compensate the lack of training data, carefully choosing the model architecture and applying mixup to cross-corpus data augmentation.

All existing studies on acoustic H-M AD were conducted using a similar setup. This experimental setup consists of a user talking either to a interlocutor or to an SDS within the same conversation. Regarding the addressee patterns, most analyses of the speech characteristics capture clear differences between human-directed and machine-directed utterances [30,31].

It furthermore has to be mentioned that the so far used data sets capture the capabilities of actual human-computer interaction systems, which are significantly lower compared to humans’ communicational capabilities. Neither the complexity of the utterances, the ability to process ellipses/topic changes, nor the understanding of emotional or laughter speech is similar between them [32,33].

The existing studies were designed in such a way that the system and the interlocutor always played different dialogue roles, e.g., the system tended to interact with the user passively, only responding to the user’s queries, while the interlocutor could behave actively, initiating his or her interaction with the user [9,14]. Moreover, the H-M interaction was more problem-oriented than the H-H one [34,35]. The various dialogue roles resulted in essential lexical differences between the H-M and the H-H dialogue domains [31,36]. Furthermore, the effect of a visible counterpart arose since the user could see both conversational partners simultaneously. The authors of [37] investigated this effect and noted that the way people talked to a technical system depended on how it looked like, i.e., whether it had a human-like appearance or not. As a result, these factors could potentially influence speakers’ behaviour, leading to more obvious addressee patterns. Regarding H-M dialogues, humans usually have some negative experience in their daily living to adapt themselves to the limitations of technical systems. This results in a slower, more pronounced but less modulated way of speaking with a limited vocabulary and being mostly non-spontaneous [15,38]. Given these considerations, we claim that a real benchmark data set for AD systems should take into account future developments where H-H and H-M dialogues are getting more similar due to the growing capabilities of future technical systems.

The authors of [23] designed RBC to eliminate most of the aforementioned factors influencing the dialogue complexity and therefore the addressee behavior of the participant. The only remaining factor was the speaker’s explicit awareness of their interlocutor’s nature (technical system or human being). As it is hardly feasible (a) to design a realistic and trustworthy technical system with the same conversational capabilities as humans and (b) to conduct a convincing experiment where the participants behave naturally and use the same conversational style towards the system and another human, the authors developed a data set where the complexity of the H-H dialogues is adjusted towards the limited capabilities of existing technical systems in order to obtain equal conditions for both types of conversations. In this corpus, described in Section 4.3, a user solves a certain task with a human agent, and then the user accomplishes a similar task with an SDS so that the agent and the system play the same dialogue role with identical dialogue complexity. The effect of a visible counterpart does not appear since the interlocutors do not see each other during a phone call. The complexity-identical setup should be considered to be a diagnostic experiment enabling a better understanding of the acoustic H-M AD problem and not as an individual problem statement. The main advantage of this corpus is that it represents a benchmark data set with a similar dialogue complexity.

## 3. Methods

**Figure 1:**
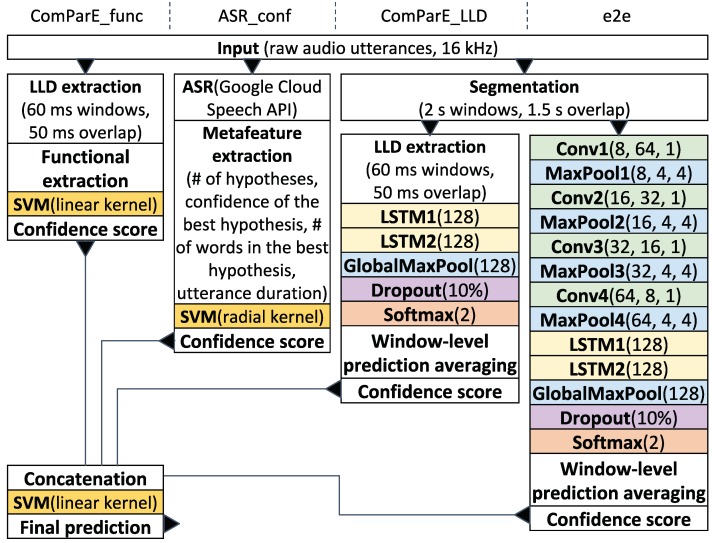
Proposed classifier architecture. Notation of convolutional layers: layer_name(n_units, filter_size, stride). Other network layers: layer_name(n_units).

To improve the classification performance, we employ an ensemble method where sophisticated classifiers are fused as depicted in Figure 1. Furthermore, we apply data augmentation to increase the available amount of training data. As input, the ensemble receives the originally segmented utterances as they were defined by the authors of the corpora. Next, we segment the utterances into context windows of various lengths for two of our models: ComParE_LLD and e2e. The windows containing only silence are excluded from consideration. See Section 5.3 for more details on the segmentation.

### 3.1. Classifier Architecture

The first model (*ComParE_func*) is similar to the baseline classifier from [23] in the sense that it is also based on a support vector machine (SVM) with a linear kernel. However, our model processes an essentially extended attribute set of 6373 acoustic functionals extracted at the utterance level using the Interspeech 2013 ComParE feature configuration [39] of the openSMILE toolkit [40]. The ComParE features have already been applied to various AD problems [20,22,28].

The second model (*ASR_conf*) is based on an SVM with a radial kernel. As input, this model receives a vector of metafeatures obtained from an automatic German speech recogniser (Google Cloud ASR [41]) and representing an ASR confidence. The core idea of applying such models to acoustic H-M AD is connected with the observation that ASR-directed speech tends to match speech recognition patterns (acoustic and language models) better compared to human-directed speech since people usually simplify and vocally emphasise their speech directed to the system, see [19] for details. ASR metainformation may also be used for improving the overall performance of an SDS [42].

The third model (*ComParE_LLD*) is a recurrent neural network containing two stacked LSTM layers followed by a global max pooling, a dropout, and a softmax layer. As input, this model receives the same sequence of low-level descriptors (LLDs) used for computing the ComParE functionals [39] for the ComParE_func model. Each sequence element is a vector of 130 LLDs extracted for a sliding time window of 60 ms with an overlap of 50 ms.

The fourth model (*e2e*) is an end-to-end version of the previous one: the LLD extractor is replaced by a convolutional component containing a stack of convolutional and pooling layers. This component was taken from the five-layer SoundNet architecture [43] and modified for our needs: the fifth convolutional layer was cut off, and the number of filters and their sizes were scaled in accordance with the available amount of our training data and the input signal resolution respectively. Rectified linear unit (ReLU) is used as an activation function after each convolutional layer. Batch normalisation is applied between each convolution and activation. For the other models mentioned above, we use statistical corpus normalisation by bringing the handcrafted features to zero mean and unit variance. A similar convolutional recurrent neural network was already applied to AD [20] and emotion recognition [44]. Our end-to-end network has considerably fewer parameters (0.3 M) compared to the network from [21] (1 6M).

For both ComParE_LLD and e2e networks, the loss function is computed as binary cross-entropy and Adam [45] is applied as a weight optimisation algorithm. The networks are trained for 100 epochs with a batch size of 32 examples. The initial learning rate is optimised on a development set and then divided by 10 if there is no performance improvement observed for the past 10 epochs on the development set. We make checkpoints, saving the current model weights at each epoch and using the best checkpoint as the resulting model according to its performance on the development set. In contrast to [27], we also optimise the SVM complexity parameter *C* of the ComParE_func model on a development set similarly to [20]. Therefore, some results obtained with this model in Section 5.1 and Section 5.2 are slightly higher than those reported in [27].

The confidence scores from all the models are concatenated and fed to a metamodel using a linear SVM as depicted in Figure 1. This model is trained on a development set, while the low-level classifiers are trained on a training set. As a performance metric, unweighted average recall (UAR) is calculated. The neural networks were developed using TensorFlow [46], the other models were designed in RapidMiner [47].

### 3.2. Data Augmentation

Data augmentation helps us to combat overfitting that is a critical issue for neural networks. For this purpose, we apply a simple yet efficient approach called *mixup* [25]. This method regularises our model by encouraging it to behave linearly in the vector space between seen data points. We propose a novel Algorithm 1 that combines mixup and multitask learning strategies and allows our model to learn data from several similar corpora in a smooth manner [27].
**Algorithm 1:** One training epoch of the proposed algorithm based on mixup.
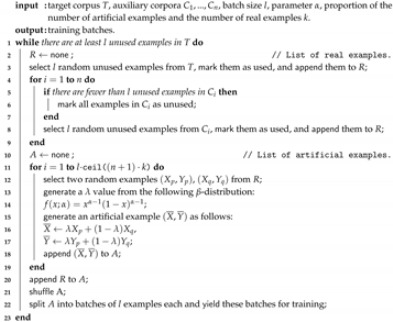


The β-distribution is defined by a parameter α that lies within the interval (0,∞) and determines the probability that our generated example lies close to one of real examples. We noted that the algorithm gave adequate results with α values lying in the interval (0,1), i.e., when each generated example biased toward one of real examples. Another algorithm parameter *k* defines the proportion of the number of artificial examples that should be generated and the number of real examples. The algorithm does not result in any considerable delays during the training process since artificial examples are generated batchwise. The parameters α and *k* are optimised on a development set.

Algorithm 1 mixes real examples regardless of their class and corpus. As a result, the labels of artificial examples are soft targets that partially resolves the problem of imbalanced training data. According to our experiments, mixing only those examples belonging to one class or to one corpus does not improve the classification performance in comparison with the current version of the algorithm.

## 4. Corpora

Our experiments were conducted on three data sets: Voice Assistant Conversation Corpus (VACC) [24], SmartWeb Video Corpus (SVC) [9], and RBC [23]. The speech data of all three corpora was uttered in German and segmented into utterances; each utterance was manually annotated as human- or machine-directed. The characteristics of the used data sets and training, development, and test partitions are given in Table 1.

### 4.1. Voice Assistant Conversation Corpus

VACC (Voice Assistant Conversation Corpus is available from their authors (ingo.siegert@ovgu.de) for research purposes upon written request.) was collected within real spoken conversations between a user, a confederate, and an SDS (Echo Dot Amazon Alexa) [24]. The recordings took place in a living room-like surrounding so that the participants could get into a more informal communicational atmosphere compared to a laboratory setting. During each experiment, a user was solving various tasks with Alexa, e.g., making appointments or answering quiz questions. While solving the tasks, the user was cooperating with a confederate, e.g., discussing possible appointment dates or answers to a quiz question. The confederate was only assisting the user and has never talked to Alexa directly. A small part of the machine-directed utterances in the VACC corpus starts with the wake-word (“Alexa”). Thus, we ran our experiments on the unchanged VACC data and also excising the trigger-word.

### 4.2. SmartWeb Video Corpus

SVC (SmartWeb Video Corpus is available from the catalogue of the European Language Resources Association (ELRA). was collected within large-scale Wizard-of-Oz (WoZ) experiments and consists of realistic spoken conversations between a user, a confederate, and a mobile SDS in the context of a visit to the Football World Cup 2006 [9]. A user was carrying a mobile phone, asking questions of different categories (world cup schedule and statistics, transport, sightseeing, and also open-domain questions), and discussing the obtained information with a confederate, who has never talked to the SDS directly. The recordings were conducted in public places and therefore contain a lot of background noise. For compatibility with the other corpora, we consider the two-class SVC problem and the partitions introduced in [9]. However, there was no development set specified, and therefore we use 20% of the speakers from the SVC training set as a development set.

### 4.3. Restaurant Booking Corpus

RBC (Restaurant Booking Corpus is available from the authors (ingo.siegert@ovgu.de) for research purposes upon written request.) is one of the first corpora of its kind, aimed at investigating the H-M AD problem from a purely acoustic point of view [23]. The corpus consists of phone calls between a customer and a restaurant booking service that is represented by a human agent or by a SDS simulated by a WoZ technique. The participants being recorded in a living room-like surrounding were asked to book tables in restaurants, taking into account several requirements, such as desirable date, time, and cuisine, possibility to sit outside, etc. Each participant took part in several experimental sessions: talking to a human interlocutor and talking to an SDS. The participants were informed beforehand that they would talk to an artificial conversational agent and to a human agent, which were clearly distinguishable by voice. The booking requirements were set to be different for each experimental session. The statements given by the human agent and the SDS were identical, and therefore the H-M and H-H dialogues had the same complexity level. This experimental setup thus eliminates most of the factors that may influence the speakers’ addressee behaviour implicitly: different dialogue roles of the addressees, the effect of a visible counterpart, the lexical content, the dialogue domain, and the different dialogue complexity. The only factor that remains is the people’s explicit awareness of their interlocutor’s nature (technical system or human being). We realise that RBC represents a compromise in achieving complexity-identical H-H and H-M dialogues; the limitations applied—simple conversations with the human conversational partner, no parallel interaction with the human and technical system—were needed to allow a plausible experimental setting for the participants.

## 5. Results and Discussion

In Section 5.1, we conduct several experiments with feature selection using the ComParE_func model. Next, we carry out a series of cross-corpus experiments with the same model in Section 5.2. Section 5.3 contains experiments with the ComParE_LLD model on various acoustic context lengths. The experiments reported in the latter three subsections do not use mixup. Finally, we combine all four models with Algorithm 1 and compare the obtained classification performance with the baseline and humans’ performance on RBC in Section 5.4.

### 5.1. Feature Selection

**Figure 2:**
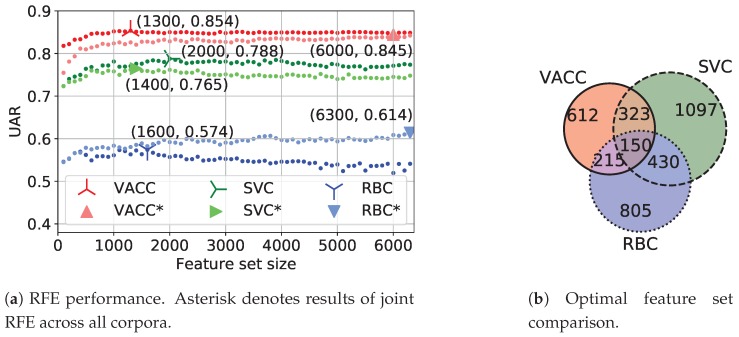
Feature selection results.

First, we want to determine the degree of similarity between our corpora in the sense of common relevant acoustic features and apply recursive feature elimination (RFE) in combination with the ComParE_func model for this purpose. The coefficients of the normal vector of the SVM are used as attribute weights similarly to [28]. Figure 2a demonstrates RFE curves obtained by applying ten-fold leave-one-speaker-group-out (LOSGO) cross-validation on various corpora without their test sets. The resulting performance is calculated as UAR and averaged over all folds for each reduced feature set. One set of curves visualises RFE on individual corpora; another set of curves marked with an asterisk reflects joint RFE across all three corpora. To obtain the latter curves, we compute a single weighting list on all the corpora merged in equal proportions and use a single ComParE_func model, which is also trained on all the corpora, to calculate the classification performance on them. We consider a feature set to be optimal if further RFE iterations worsen the classification performance stably. Intersections of three optimal feature sets, each of which was obtained on a random fold by RFE on individual corpora, are depicted in Figure 2b. The representative acoustic functionals vary considerably: VACC, SVC, and RBC have only 1300, 2000, and 1600 relevant features out of 6373 respectively, while having only 150 features in common. However, these common functionals concern each of the 130 ComParE LLDs in some form [39], i.e., the feature selection is reduced to the selection of the optimal functionals for each LLD. For this purpose, we apply the ComParE_LLD model in Section 5.3 and Section 5.4. Due to its recurrent architecture, this model can learn its own, "custom" functionals from the time-continuous LLD sequences for the particular task.

According to the joint RFE experiments (marked with an asterisk in Figure 2a) across all three corpora, the entire set of the 6373 ComParE functionals should be used for joint classification since the performance on VACC and RBC starts dropping directly after the first RFE iterations and the performance on SVC does not reach the values of individual RFE. In particular, it can be seen in Figure 2a that the performance of the entire functional set on RBC is always higher than the performance of any of its subsets. In other words, the 150 common functionals visualised in Figure 2b are important but not enough to classify RBC utterances reliably.

### 5.2. Leave-One-Corpus-Out Experiments

**Figure 3:**
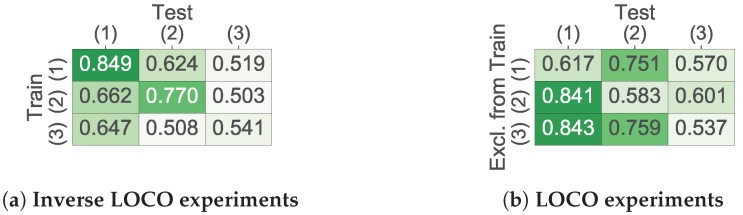
Cross-corpus experiments. All results are presented in terms of UAR. Corpora: (1) VACC, (2) SVC, (3) RBC.

Second, we conduct leave-one-corpus-out (LOCO) and inverse LOCO experiments with the ComParE_func model to estimate its behaviour on unknown corpora. In both cases, the entire ComParE feature set is used without RFE and the model is trained and tested on the corresponding partitions from Table 1. Results of both experimental series are depicted in Figure 3. Inverse LOCO means that the classifier is trained on one corpus (rows in Figure 3a) and individually tested on each of the corpora (columns in Figure 3a). LOCO means that the classifier is jointly trained on all corpora but one (rows in Figure 3b) and individually tested on each of the corpora (columns in Figure 3b). Let us denote the matrix from Figure 3a as *A*, the matrix from Figure 3b as *B*, and their elements as $a_{i,j}$ and $b_{i,j}$ respectively. $a_{1,2}$ and $a_{2,1}$ are considerably greater than the other off-diagonal elements of *A*, demonstrating a clear relation between VACC and SVC. The other off-diagonal elements of *A* are close to a random-choice UAR of 0.5. However, $a_{3,1}$ is greater than $a_{3,3}$, meaning that the model trained on RBC performs better on VACC than on the RBC test set. In other words, training on the complexity-identical setup allows the model to capture true acoustic patterns that are also typical for the classical H-M AD setup. However, the opposite statement does not work due to collateral factors influencing the users’ behaviour in the latter setup only and which cause the model to overfit. SVC and RBC do not exhibit such a relationship probably due to the difficult acoustic conditions of SVC (it is the only corpus containing not only indoor recordings but also outdoor recordings made by a distant-microphone). Another remarkable observation is $b_{1,3}$ and $b_{2,3}$ being greater than $a_{3,3}$ and meaning that merging RBC and another corpus improves the classification performance on RBC compared to using the RBC training data only. This positive result motivates us to apply Algorithm 1 rather than just merging examples from different corpora.

### 5.3. Experiments with Various Acoustic Context Lengths

**Figure 4:**
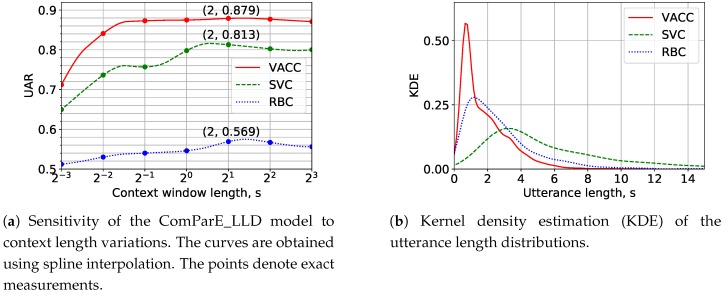
Experiments with various context window lengths.

Next, we determine the optimal acoustic context length for H-M AD. The segmentation into context windows allows us to increase the number of examples and partially offsets the lack of training data for neural networks. Context windows are extracted with an overlap of 75% of their length. The windows containing only silence are excluded from consideration. We suppose the most important vocal addressee patterns to appear at short context lengths of around 1 s and therefore use a logarithmic scale depicted in Figure 4a. For each context length, the ComParE_LLD model is examined on the LOSGO cross-validation. The resulting UAR is averaged over all folds. We confine to a context length of 2 s in our experiments with the ComParE_LLD and the e2e model in Section 5.4 since this value is optimal for all three corpora, though the maximum of the KDE values of their utterance length distributions depicted in Figure 4b are around 1 s for VACC and RBC and around 3 s for SVC. The differences between the average utterance lengths appeared due to various interaction complexity levels supported by the systems. Two-second speech fragments were also shown to be sufficient for H-M AD in English [14].

### 5.4. RBC Data Augmentation

Both SVM- and neural network-based models show considerably lower performance values on RBC compared to the other two corpora. In contrast to [21,22], we do not have an Amazon-scale amount of training data, though data augmentation helps us to combat this problem. We conduct a series of experiments, simply merging RBC with the other corpora or doing the same in combination with Algorithm 1. Results of these experiments computed on the partitions from Table 1 are given in Table 2. Alongside with UAR, unweighted average precision (UAP) is calculated. We noted that applying several regularisation techniques, e.g., mixup, Gaussian noise, and high dropout ratios, at once eliminated their positive effect, and therefore we use low dropout ratios and no additional noise when Algorithm 1 is activated. The performance values obtained with the models (1)–(4) on the RBC development set demonstrate that these classifiers benefit from merging RBC and another corpus and especially from merging RBC and VACC. According to the LOSGO cross-validation and a *t*-test with a significance level of 0.05, these classification improvements are significant. However, merging all three corpora is not the most effective training strategy due to a low proportion of RBC in the augmented training data: sometimes the model starts neglecting RBC in favour of the other corpora. Mixup does not provide any significant performance improvement for the SVM-based models (1) and (2) on the RBC development set due to their simple architectures that do not require regularisation. Mix(RBC + VACC) and mix(RBC + SVC) significantly improve the performance of the ComParE_LLD model (3) on the RBC development set, while significantly worsen the performance of the e2e model (4) compared to merging the same corpora without mixup. This result may be explained by the nature of mixup: when applied to raw audio signal, the method just overlaps two audio files and does not differ from noise augmentation essentially. Furthermore, it may cause the cocktail party effect confusing the model. The aforementioned differences between the performance values of individual classifiers remain for the RBC test set. However, the performance values on this set are slightly higher than the corresponding ones on the RBC development set as the former values were obtained using the RBC development set in addition to the training sets. The following model configuration has been chosen for metafusion: ASR_conf(RBC + VACC) + ComParE_func(RBC + VACC) + ComParE_LLD(mix(RBC + VACC)) + e2e(RBC + VACC). The metamodel (5) demonstrates a UAR of 0.628 and a UAP of 0.632 on the RBC test set. We compare these results with the performance of the baseline classifier (6) and with the non-native (7) and native listeners’ (8) classification performance, applying the LOSGO cross-validation and a *t*-test with a significance level of 0.05. Our metaclassifier significantly surpasses the baseline classifier and both groups of human listeners on RBC in terms of both UAR and UAP. Each individual classifier of the proposed metamodel also significantly surpasses the baseline classifier and the non-native listeners and performs at the native listeners’ level. The baseline classifier and the perceptual experiments with the human listeners are described in detail in [23].

## 6. Conclusions

Our experiments revealed a pragmatic difference between the classical and the complexity-identical H-M AD scenario. On the one hand, the necessity to choose between a human and a machine interlocutor within the same conversation appears to be a strong motivation for users to change their manner of speech in order to emphasise the desirable addressee. On the other hand, users demonstrate ambiguous acoustic addressee patterns in the absence of collateral factors, such as different dialogue roles of addressees, the effect of a visible counterpart, different lexical content, and different dialogue domains of human- and machine-directed utterances.

Thus, acoustic H-M AD in complexity-identical scenarios turned out to be a significantly more challenging problem than classical acoustic H-M AD. Acoustic changes that take place in classical H-M AD include acoustic variations exhibited in complexity-identical H-M AD but are not confined to them. Even native listeners can hardly resolve addressee ambiguity in complexity-identical scenarios. Non-native listeners completely fail to determine the addressees in RBC so that any of our models outperforms or at least keeps up with them. With our classification architecture, we managed to surpass the native listeners’ performance on RBC. The use of the out-of-domain data from other corpora alongside with RBC is highly beneficial in this case, though the other two corpora were designed for classical H-M AD scenarios. Mixup helps us to regularise complex models, i.e., neural networks operating on handcrafted features.

Excising the wake-word (here “Alexa”) from the VACC data may have caused a problem since it may result in utterances that no longer have a natural prosody, and artefacts from the data manipulation could give inadvertent cues to the machine learning system. However, our experiments with excising the wake-word do not influence the results significantly. This can be explained as follows. First, we do not use the ASR output directly for classification. Second, only a small part of the machine-directed utterances in VACC starts with the wake-word. Third, the artefacts that appear after excising the wake-word still have the same meaning for our models as the wake-word itself.

The general applicability of the proposed architecture to different languages, acoustic conditions, and applications has already been shown, e.g., in [27,48], thus it can be assumed that the proposed approach is also usable for a broader AD application. An in-depth analysis with different data sets similar to RBC has to be conducted. However, this has not been possible so far due to the absence of a suitable data set.

## Figures and Tables

**Table 1:** General characteristics of the analysed data sets and their utterance-level labelling. Each utterance was manually annotated as human- (H) or machine-directed (M).

Corpus	VACC			SVC			RBC		
**Set**	**Train**	**Dev**	**Test**	**Train**	**Dev**	**Test**	**Train**	**Dev**	**Test**
M	1809	501	1493	546	90	442	752	142	558
H	862	218	756	557	135	423	368	86	328
# of utterances (# of speakers)	2671 (12)	719 (3)	2249 (10)	1103 (48)	225 (10)	865 (41)	1120 (16)	228 (3)	886 (11)
	5639 (25), 2:50:20 s			2193 (99), 3:27:35 s			2234 (30), 1:39:17 s		

**Table 2:** Classification performance on RBC in terms of UAR (upper value) and UAP (lower value). Each of the columns (1)–(4) has two subcolumns. The left subcolumn contains performance values computed on the RBC development set, the right subcolumn contains performance values obtained on the RBC test set. The other performance values are computed on the RBC test set. A bold value denotes the best result in the column/subcolumn. The underlined bold value shows the best result overall. Classifiers: (1) ASR_conf, (2) ComParE_func, (3) ComParE_LLD, (4) e2e, (5) metamodel, (6) baseline classifier, (7) non-native listeners, (8) native listeners. Configuration of the model (5): ASR_conf(RBC + VACC) + ComParE_func(RBC + VACC) + ComParE_LLD(mix(RBC + VACC)) + e2e(RBC + VACC). The classifiers (1) and (2) use entire utterances. The classifiers (3) and (4) use context windows of 2 s. The “+” mark denotes a simple merger of several corpora. The “mix” mark means that Algorithm 1 is applied.

Data Augmentation Method	(1)		(2)		(3)		(4)		(5)	(6)	(7)	(8)
RBC	0.539	0.550	0.533	0.541	0.548	0.556	0.552	0.570	**0.628**	0.539	0.544	0.596
	0.643	0.659	0.565	0.589	0.559	0.572	0.558	0.578	0.632	0.540	0.544	0.591
mix(RBC)	0.533	0.552	0.526	0.540	0.532	0.545	0.529	0.548	-	-	-	-
	0.635	0.657	0.568	0.581	0.546	0.566	0.548	0.568	-	-	-	-
RBC + VACC	**0.577**	**0.590**	**0.579**	**0.601**	0.584	0.598	**0.591**	**0.609**	-	-	-	-
	0.617	0.630	0.619	0.638	0.599	0.611	0.606	0.622	-	-	-	-
mix(RBC + VACC)	0.574	0.588	0.577	0.598	**0.604**	**0.620**	0.548	0.569	-	-	-	-
	0.615	0.628	0.616	0.635	0.613	0.631	0.567	0.581	-	-	-	-
RBC + SVC	0.546	0.567	0.549	0.570	0.547	0.567	0.563	0.587	-	-	-	-
	0.585	0.598	0.580	0.599	0.556	0.577	0.575	0.598	-	-	-	-
mix(RBC + SVC)	0.554	0.569	0.546	0.569	0.563	0.582	0.513	0.529	-	-	-	-
	0.581	0.597	0.584	0.597	0.576	0.591	0.537	0.554	-	-	-	-
RBC + VACC + SVC	0.500	0.502	0.574	0.600	0.539	0.552	0.545	0.554	-	-	-	-
	0.578	0.590	0.619	0.632	0.541	0.560	0.547	0.568	-	-	-	-
mix(RBC + VACC + SVC)	0.500	0.500	0.576	0.595	0.535	0.554	0.513	0.531	-	-	-	-
	0.573	0.591	0.605	0.624	0.555	0.569	0.540	0.551	-	-	-	-
